# Relationship Between Endemic and Invasive Frogs on Grenada

**DOI:** 10.1002/ece3.72889

**Published:** 2026-01-11

**Authors:** Billie Harrison, Julie Beston

**Affiliations:** ^1^ University of Wisconsin Milwaukee Wisconsin USA

**Keywords:** amphibian declines, *Eleutherodactylus johnstonei*, Grenada frog, invasive species, island endemic, *Pristimantis euphronides*

## Abstract

Invasive species, loss of habitat, and climate change are just some of the many threats accelerating biodiversity loss, and understanding their impacts on endangered species is key to implementing effective conservation. The endemic Grenada frog ( *Pristimantis euphronides* ) is found only in high elevation cloud forests, habitat that is being invaded by the introduced Lesser Antillean frog ( *Eleutherodactylus johnstonei* ) and threatened by climate change. Between 2004 and 2020, our field team surveyed three key sites in the central highlands of Grenada to monitor populations of both frogs. We used generalized linear models and Spearman's rank analysis to evaluate the effects of site and invasive frog relative abundance on the endemic frog. Although the relative abundances of the two species were negatively correlated overall (*ρ* = −0.501, 95% CI: (−0.660, −0.300)), the relationship between them was weakly positive in the model that included site as a covariate. The two species appear to respond similarly to environmental fluctuations at local scales, but the negative overall correlation implies that competition with the Lesser Antillean frog may have affected the distribution of the Grenada frog across the island. Grenada frogs were much more abundant than Lesser Antillean frogs at the highest elevation site, while the reverse was true at the lower sites. If higher elevation sites are indeed acting as refugia for the Grenada frog from its invasive competitor, the effects of climate change at those high elevation sites will likely be critical to the future of the species.

## Graphical abstract

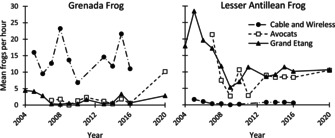

## 1 Introduction

Amphibians serve in many critical capacities within our ecosystems, and yet they face startling imperilment, extreme even for our current era of mass extinction. The global decline in amphibians is unprecedented among other taxa throughout human history (Stuart et al. 2004), and assessing the confluence of threats facing amphibians severely complicates conservation action planning. While the challenges they face are not unique, they are particularly susceptible as a consequence of their unique physiology and life histories. Their permeable skin and lack of amniotic membranes leave them vulnerable to desiccation and reproductive failure in the face of changing hydrologic regimes. They have somewhat primitive immune systems and may be more susceptible to emerging pathogens than other tetrapods. Indeed, the fungal pathogen *Batrachochytrium dendrobatidis* has been blamed for many of the declines (Berger et al. 1998; Bosch et al. 2001; Daszak et al. 2003; Lips et al. 2003; Muths et al. 2003).

Among endangered amphibians, the endemic Grenada frog ( *Pristimantis euphronides* ) is particularly vulnerable to extinction. Among the threats it faces is the non‐native Lesser Antillean frog ( *Eleutherodactylus johnstonei* ), which was introduced to Grenada in 1885 (Barbour 1914) and has successfully invaded much of the island. The impacts of invasive species tend to be disproportionately high on islands compared to continental locations (Russell et al. 2014), and there are several characteristics that may allow the nonnative frogs to outcompete Grenada frogs (Ortega et al. 2005). Many invasive species can thrive across broad environmental conditions and can establish themselves ubiquitously, while specialized island endemics are often restricted to narrow habitats. Many lowland frog species have been driven by climate change to higher elevations where they may compete with already vulnerable high‐elevation endemics (Huey et al. 2012). Both species exhibit parental care of clutches laid on moist surfaces. However, while female Grenada frogs are responsible for tending to the egg mass to prevent desiccation, this job tends to fall to the male Lesser Antillean frogs (Bourne 1998). That allows female Lesser Antillean frogs to replenish the metabolic stores needed to produce another clutch of eggs. In addition, Lesser Antillean frogs have relatively high tolerance of desiccation and extreme heat (Pough et al. 1977). Their increased reproductive capacity and environmental tolerance may give them a competitive advantage over Grenada frog, especially in the drier, warmer forests found at lower elevations.

Another major concern for these imperiled frogs is habitat loss. The Grenada frog is believed to be restricted to pristine forests above 300 m (Henderson and Berg 2006). Land conversion for agriculture has destroyed 85% of the forest cover once found on Grenada (Henderson and Powell 2018), and climate change is likely to drive further loss of the high elevation forests Grenada frogs rely on. Climate change is also likely to exacerbate any negative effect of invasive Lesser Antillean frogs that are more tolerant of warm, dry conditions. Indeed, many lowland frog species have responded to climate change by shifting to higher elevations where they can compete with already compromised high‐elevation endemics (Huey et al. 2012).

To better understand the current status and conservation needs of the Grenada frog, we examined the relative abundance of Grenada frogs and Lesser Antillean frogs at several sites on Grenada. We included sites at different elevations and with differing vegetation, and we evaluated the relationship between the relative abundance of the endemic Grenada frog and invasive Lesser Antillean frog at both regional and local scales. We expected to find a negative correlation between the population sizes of these two species due to competition between them (Eccard and Ylönen 2003; Harris 2009).

## 2 Methods

### 2.1 Study Sites

We sampled three survey sites that had known populations of Grenada frogs and typified distinct habitat types. Grand Etang is the type locality for the Grenada frog. Once a dense habitat dominated by trees and tree ferns as tall as 30 m (Beard 1949), Grand Etang became open‐canopy and bathed in sunlight in 2004 when Hurricane Ivan devastated the forests along mountain ridges. Today, ferns and tree ferns (*Cyathea* spp.) are the primary flora, but razor grasses (*Scleria* spp.) and maidenhair ferns (*Adiantum* spp.) are increasingly prominent. This site is regularly blanketed in a dense mist that condenses in the cooler air of Grenada's mountain ranges when trade winds carry moisture from the Atlantic Ocean up the slopes.

Despite being contiguous with the ravaged Grand Etang National Park, the Avocats site is located between two mountain ridges that buffered it from the full impacts of Hurricane Ivan. Consequently, Avocats has remained a closed canopy forest. This topography also limits the presence of mist that so often hangs over the other two sites. Tree ferns and razor grasses are rare; trees, bamboo ( *Bambusa vulgaris* ), and heliconia are common.

The third site, Cable and Wireless, is thought to be a Grenada frog stronghold and one of a few locations on the island where the slope is too extreme to be cleared for agricultural use (Henderson and Berg 2006; Sander et al. 2003). Before Hurricane Ivan, the landscape was dominated by broadleaf trees (*Myristica* sp. and *Swietenia* sp.) and shrubs, tree ferns, and other ferns. Currently, the site is covered in saber grass (*Scleria* sp.) and ferns with some saplings. With the elevation of this ridge being 175 m higher than Grand Etang, the air is colder, a dense mist often hangs in the air, and rain falls regularly.

### 2.2 Field Methods

Since 2004, B.H. and field crews have monitored the three primary survey sites by walking timed transects. Three 100‐m transects were established at each site. Two observers covered a 100‐m transect in 30 min, one searching each side of the transect, resulting in 1 h of search effort per transect per survey. The visible areas within 2 m of the transect were visually scanned for frogs. Surveyors also listened for nearby frog calls. We recorded the species of each animal and, if possible, its age class, sex, perch type, and perch height. Frogs were not handled or disturbed. Surveys took place in the dry season (January–March), early wet season (May–June), and late wet season (October–November), although not every season was surveyed every year.

### 2.3 Data Analysis

We estimated the average number of Grenada frogs and Lesser Antillean frogs observed per hour of search effort across all transects at each site in each season of each year it was surveyed. This allowed for a standardized measure of relative abundance despite some variability in total search effort at each site across surveys. We then calculated Spearman's rank correlation coefficient between average Grenada frogs and Lesser Antillean frogs per hour.

We then fit a suite of 8 nested candidate models for Grenada frogs per hour to explore the potential effects of location, timing, and invasive frogs. Every candidate model included site as a covariate, and we considered models that included combinations of season, year, and Lesser Antillean frogs per hour. We treated year as a factor variable to allow for stochastic annual fluctuations. We fit models using generalized linear regression in R (R Development Core Team 2011) and used AICc for model selection (Akaike 1973).

## 3 Results

**FIGURE 1:**
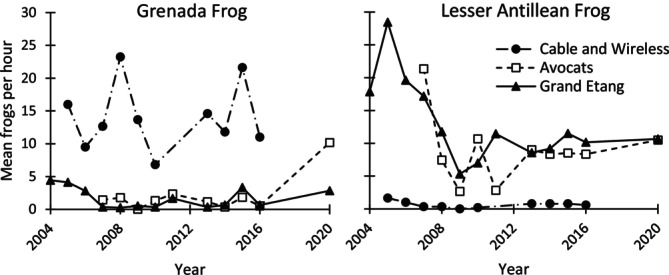
Changes in relative abundance of Grenada and Lesser Antillean frogs during dry season surveys at three sites in Grenada, 2004–2020.

We observed 7028 frogs during 447 h of surveys between February 2004 and January 2020, with an average (95% CI) of 15.88 (14.25, 17.19) total frogs per hour. The average number of Grenada frogs per hour was 5.92 (4.44, 7.40), while that of Lesser Antillean frogs was 9.96 (8.30, 11.61). Observations per hour for both species varied considerably, from a minimum of zero individuals to a maximum of 32 Grenada frogs in a single hour in 2008 at Cable and Wireless and 61 Lesser Antillean frogs in 2005 at Grand Etang. The average dry season observations of Lesser Antillean frogs seemed to decrease between 2005 and 2008 at Grand Etang and Avocats, but no consistent trends were apparent for either species across the duration of the study (Figure 1). Overall, the Spearman's rank correlation between Grenada frogs and Lesser Antillean frogs was −0.501 (−0.660, −0.300).

**FIGURE 2:**
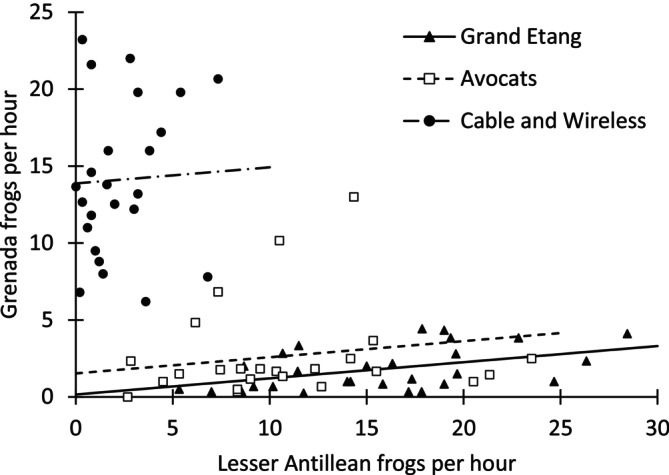
Observations (points) and model predictions (lines) of Grenada frogs per hour vs. Lesser Antillean frogs per hour at three study sites.

The top‐ranked model was the simplest we fit, with site as the only explanatory variable (Table 1). However, the ΔAICc for the model that also included Lesser Antillean frogs per hour was less than two, suggesting it was comparable in support. Because of our interest in the effect of invasive frogs, we present results for this second‐ranked model that included them. The estimated Grenada frogs per hour in the absence of Lesser Antillean frogs was similar at Grand Etang, 0.157 (−2.665, 2.979), and Avocats, 1.528 (−0.676, 3.732). The estimate for Cable and Wireless was considerably higher, at 13.873 (12.455, 15.291). Lesser Antillean frogs per hour exhibited a weak positive association with Grenada frogs per hour within sites, with a coefficient of 0.105 (−0.056, 0.266) (Figure 2).

**TABLE 1:** Model selection results for Grenada frogs per hour as a function of study site (site), Lesser Antillean frogs per hour (Lesser Antillean frog), season of observation (season; dry, early wet, or late wet), and/or year of observation (year; as a factor variable).

Model	Parameters	ΔAICc
Site	4	0.000
Site + Lesser Antillean frog	5	0.588
Site + season	6	4.374
Site + Lesser Antillean frog + season	7	5.231
Site + year	18	8.783
Site + Lesser Antillean frog + year	19	11.523
Site + season + year	20	15.637
Site + Lesser Antillean frog + season + year	21	18.910

## 4 Discussion

The negative overall correlation between Grenada frog and Lesser Antillean frog relative abundances is consistent with expectations for two species in competition with one another (Eccard and Ylönen 2003; Harris 2009), but that is not the only possible explanation. The linear models revealed that the overall negative correlation could be explained by differences among the sites. Grand Etang and Avocats had low Grenada frog abundance and high Lesser Antillean frog abundance, and the opposite was true at Cable and Wireless. It is difficult to assess whether this pattern resulted from direct competition or simply reflects differences in habitat use of these two species.

Lesser Antillean frogs take advantage of habitat disturbed by anthropogenic land use and natural catastrophes that tend to reduce native frog populations (Kaiser 1997). Thus, the destruction associated with Hurricane Ivan could explain the relatively high abundance of Lesser Antillean frogs and low abundance of Grenada frogs at Grand Etang. However, that explanation is not consistent with our observations of similarly high Lesser Antillean frog abundance at Avocats, where Ivan had little impact, and low abundance at Cable and Wireless, where trees and shrubs were largely replaced by grass after the hurricane. It may be that the local conditions at the higher elevation Cable and Wireless site provide a competitive advantage to Grenada frogs that keeps the population size of the invaders low. Avocats is at a similar elevation to Grand Etang, and thus offers warmer, drier conditions that may favor the Lesser Antillean frog and allow them to suppress population growth of Grenada frogs.

Despite the overall negative association, we found a slight positive relationship between the two species when differences among sites were accounted for. This implies that both species may respond similarly to environmental fluctuations at a particular site, despite the more generalist nature of the Lesser Antillean frog. Thus, in a short‐term, local setting, conditions such as favorable weather or increased prey abundance could benefit both the Lesser Antillean and the Grenada frog, resulting in both species increasing in abundance or activity and masking the effects of competition between them at this scale. However, given the high variability of frog populations and the weak nature of the apparent link, the ecological implications of this relationship are unclear. Regardless, the apparent mismatch between the island‐wide and site‐specific relationship between Grenada and Lesser Antillean frogs does highlight the importance of doing widespread assessments, as a single site may mask the broader landscape trend.

Even with long‐term monitoring, threat assessment can be complex and often leads to further questions. There is some evidence that competition with the invasive Lesser Antillean frog limits abundance of native Grenada frogs at lower‐elevation sites and that high elevation sites may serve as refuges. Fortunately, much of the central highlands where Grenada frogs occur are protected national parks and contain steep slopes that are not inviting for agricultural development. However, global climate change will continue to shrink the high elevation habitats they prefer which will force them into tighter spatial overlap with the generalist Lesser Antillean frog that can persist across broader conditions. Global climate change will also likely lead to an increase in the frequency and severity of hurricanes that disturb habitats, often leading to an increase in invasive vegetation which does not support the Grenada frog and gives the invasive frog a stronger foothold. As a result, high elevation refugia could transition from primarily Grenada frog sites to habitat dominated by Lesser Antillean frogs. Further research including additional sites and specifically investigating potentially important environmental factors is warranted, especially given that roughly one‐third of amphibian extinctions were caused by invasive species (Blackburn et al. 2019; Falaschi et al. 2020; Stuart et al. 2004) and that island species are more heavily impacted (Spatz et al. 2017; Strayer 2010; Stuart et al. 2004).

## Author Contributions

**Billie Harrison:** conceptualization (equal), data curation (equal), formal analysis (equal), funding acquisition (lead), investigation (equal), methodology (equal), project administration (equal), resources (lead), validation (equal), visualization (equal), writing – original draft (lead), writing – review and editing (equal). **Julie Beston:** conceptualization (equal), data curation (equal), formal analysis (equal), funding acquisition (supporting), investigation (equal), methodology (equal), project administration (equal), resources (supporting), validation (equal), visualization (equal), writing – original draft (supporting), writing – review and editing (equal).

## Funding

This work was supported by the Zoological Society of Milwaukee. Milwaukee County Zoo. Safari Lake Geneva. Racine Zoological Gardens. Thomas Thorhorst Grant.

## Conflicts of Interest

The authors declare no conflicts of interest.

## Data Availability

Data supporting the findings of this study are available in [Grenada frog relationship assessment] at [https://osf.io/rp2uy/files/osfstorage], under reference numbers [h48be, pejkr].
